# ‘Cycle Thieves, We Are Watching You’: Impact of a Simple Signage Intervention against Bicycle Theft

**DOI:** 10.1371/journal.pone.0051738

**Published:** 2012-12-12

**Authors:** Daniel Nettle, Kenneth Nott, Melissa Bateson

**Affiliations:** 1 Centre for Behaviour and Evolution & Institute of Neuroscience, Newcastle University, Newcastle, United Kingdom; 2 Estate Security Service, Newcastle University, Newcastle, United Kingdom; Université de Strasbourg, France

## Abstract

**Background:**

Bicycle theft is a serious problem in many countries, and there is a lack of evidence concerning effective prevention strategies. Displaying images of ‘watching eyes’ has been shown to make people behave in more socially desirable ways in a number of settings, but it is not yet clear if this effect can be exploited for purposes of crime prevention. We report the results of a simple intervention on a university campus where signs featuring watching eyes and a related verbal message were displayed above bicycle racks.

**Methodology and Principal Findings:**

We installed durable signs at three locations which had experienced high levels of bicycle theft, and used the rest of the university campus as a control location. Reported thefts were monitored for 12 months before and after the intervention. Bicycle thefts decreased by 62% at the experimental locations, but increased by 65% in the control locations, suggesting that the signs were effective, but displaced offending to locations with no signs. The Odds Ratio for the effect of the intervention was 4.28 (95% confidence interval 2.04–8.98), a large effect compared to other place-based crime prevention interventions.

**Conclusions and Significance:**

The effectiveness of this extremely cheap and simple intervention suggests that there can be considerable crime-reduction benefits to engaging the psychology of surveillance, even in the absence of surveillance itself. Simple interventions for high-crime locations based on this principle should be considered as an adjunct to other measures, although a possible negative consequence is displacement of offending.

## Introduction

The theft of bicycles is a substantial social problem in many countries [1]. In England and Wales, for example, there were 115,905 bicycle thefts reported to police between April 2011 and May 2012 [2]. This represented an increase of 6% compared to the previous year, whereas the overall number of crimes fell by 4%. Bicycles are often stolen from on-street locations where they have been left by their owners, for example at university campuses and railway stations [3]. Although a number of initiatives for reducing bicycle theft have been experimented with, there is at present scant evidence on the effectiveness of these [3]. In this paper, we report an evaluation of the effectiveness of a simple, cheap anti-bicycle theft intervention using signs designed to evoke the psychology of being watched that was implemented at a large university campus in Northern England.

The project was motivated by two principles. The first is the need for crime prevention strategies to be evaluated quantitatively so that evidence-based policy decisions can be made. This principle has been strongly argued for in recent years [4], [5]. The minimum requirement for evaluation is crime prevalence data from both before and after the intervention, for both the locations receiving the intervention, and appropriate control locations [5]. Such evaluations, when properly described and made available, can be incorporated into systematic reviews and meta-analyses so that the general effectiveness of different kinds of interventions can be established and compared. Systematic reviews of evaluations have shown that many simple place-based crime prevention measures are effective. For example, CCTV surveillance reduces crime by an average of around 7%, with larger reductions of around 51% specifically for interventions in car parks [6]. Improved street lighting can also have beneficial effects of crime, with the average of reductions reported around 22% [5]. However, as with all place-based crime prevention strategies, there are concerns that the interventions simply displace offences to other locations rather than preventing them altogether [7]. Evidence of displacement of offending is observed in around one quarter of evaluations of place-based crime interventions, whilst around half find no evidence of it, and the remaining quarter find the opposite of displacement, diffusion of benefit [8].

The second principle was that the intervention itself should be based on contemporary behavioural science. Behavioural scientists increasingly appreciate that human decision-making can be strongly affected by manipulating the way the environment appears or information is presented [9], [10]. Because the mind often relies on fast, simple, non-conscious ‘rules of thumb’ to make decisions [11], changes to the situation that ought rationally to make little or no difference actually lead to large changes in behaviour. A good example is the ‘watching eyes’ effect. The original demonstrations of this effect showed that displaying images of eyes caused participants to behave more prosocially in laboratory contexts [12]–[14]. These laboratory findings have since been replicated and extended (e.g. [15], [16]–[18]). In addition, the watching eyes effect has been demonstrated in real-world settings such as donations to charity [19], [20], putting money in an honesty box [21], clearing litter [22], and following garbage-recycling rules [23]. The rationale for the effect is that being observed committing an act is likely to lead to social repercussions, either positive or negative, and thus it makes sense that when observed, people tailor their acts so as to be more socially desirable. The watching eyes in the studies are always just images, and thus cannot in fact observe anything. The effect occurs nonetheless, since humans have fast, automatic psychological mechanisms which have evolved to respond to all eye-like stimuli [24], and these respond to mere representations of eyes as they would to actual eyes.

Since images of watching eyes have been shown to increase compliance with social norms of contributions to an honesty box and clearing litter, we reasoned that they could potentially be effective as part of an intervention against the more serious norm-violation of stealing a bicycle. The idea that being observed reduces crime is not new. Formal surveillance such as closed-circuit television and ‘natural surveillance’ such as designing the built environment so locations are in sight of passersby are standard planks of situational crime prevention [25]. The ‘watching eyes’ effect differs from these in that it uses just *cues* of being watched, in the absence of actual observation. Although there has been considerable interest in the possibility of exploiting the effect for crime prevention purposes [26], [27], no quantitative data on the effectiveness of a ‘watching eyes’ based crime-prevention intervention have yet been presented in the literature.

One of our previous studies suggested that the effect of eyes did not depend on displaying an associated verbal message [22]. However, previous anti-theft research has suggested that displaying signs indicating verbally an awareness that theft is going on in a particular location, and an attention to it, can itself be highly effective in reducing theft [28]. Thus, we designed an intervention that would combine both watching eyes and a verbal message, by making and displaying large signs above bicycle racks. Limitations of resources and scale meant that we were not able to test which parts of the intervention were responsible for any change in thefts. For example, we did not experiment with displaying eyes without the verbal message, or the verbal message without the eyes. However, an important first step is to establish what effect if any the combination of the eyes and the verbal message had on bicycle thefts. To do this, we installed the signs for one year in three high-theft locations on a university campus, and recorded the number of notified bicycle thefts in the year before and the year after installation, for these experimental locations, and, as a control, for the rest of the campus.

## Methods

### Study Setting

**Figure 1:**
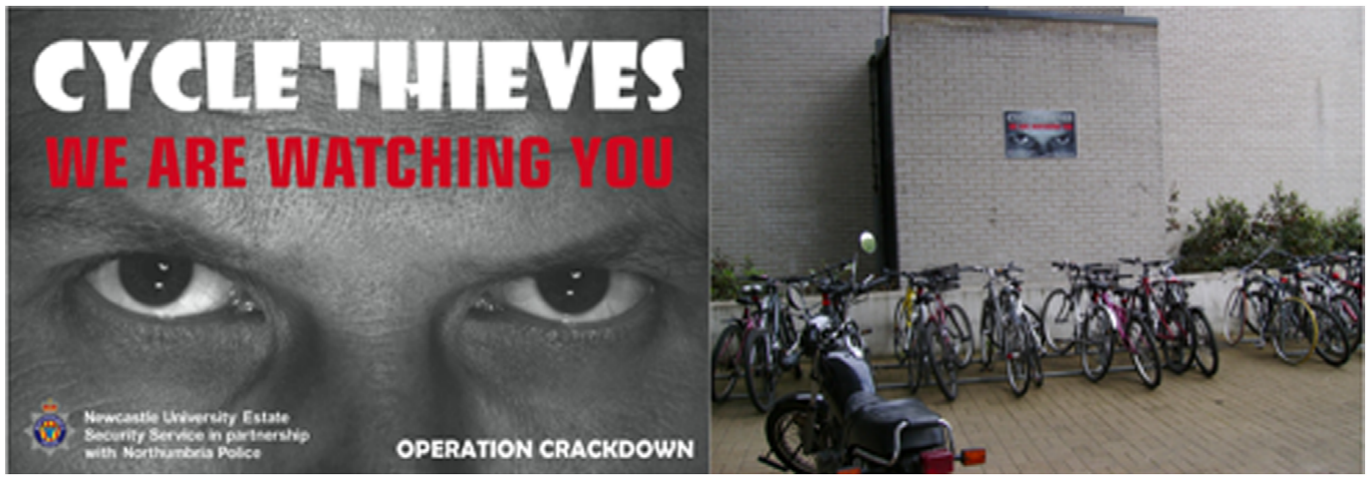
The signs used in the experimental intervention. (Left) Detail of the design. The person depicted in the sign has given written informed consent, as outlined in the PLOS consent form, to publication of their photograph. (Right) Sign *in situ* in an experimental location.

Newcastle University has a large campus within an urban area which can be freely entered by pedestrians from surrounding parts of the city at numerous points and at any time of day. The campus is covered by closed circuit television and regular foot and vehicle security patrols, but these did not change in any way over the course of the study. The use of bicycles is popular with students and staff, and these are left locked to racks and fences outside and between university buildings (see Fig. 1). There has been a persistent problem with bicycle thefts, with over 50 per year notified to the estate security service for the past several years. It is likely that there are also a considerable number of thefts that are never notified, but we have no means of estimating the prevalence of these. However, the rate of notification is likely to be fairly high, as those losing cycles require a crime number in order to be able to make an insurance claim. The estate security service maintains a database of the date and location of each notified theft.

### Experimental Intervention

We used the cycle theft database to identify three locations with particularly high rates of cycle theft. Between them, these three experimental locations had accounted for approaching half of all thefts in the previous few years. The pairwise walking distances between the experimental locations were approximately 900 m, 600 m and 400 m. The numerous other bicycle racks spread over the rest of the campus (at distances of 100–1000 m from the experimental locations) served as the control locations. Durable intervention signs measuring 90×60 cm were installed at the three experimental locations. They were sited on walls at heights of 1.5–2.5 m from the ground so as to provide maximum visibility over all the places where bicycles are left. There were three signs at the largest location and one sign at each of the other two. The signs featured a black and white image of a pair of male eyes with direct forward gaze (Fig. 1). In addition, they bore the headline ‘Cycle Thieves: We Are Watching You’, along with the name ‘Operation Crackdown’, and the logo of the local police service.

### Evaluation

The intervention signs were installed in May 2011. We used the cycle theft database to calculate the number of notified thefts in the year prior to the installation of the signs, and the year subsequent to their installation, for both experimental and control locations.

### Ethics Statement

No formal ethical approval was required for this study, since no identifiable individuals were observed in the process of conducting it.

## Results

**Figure 2:**
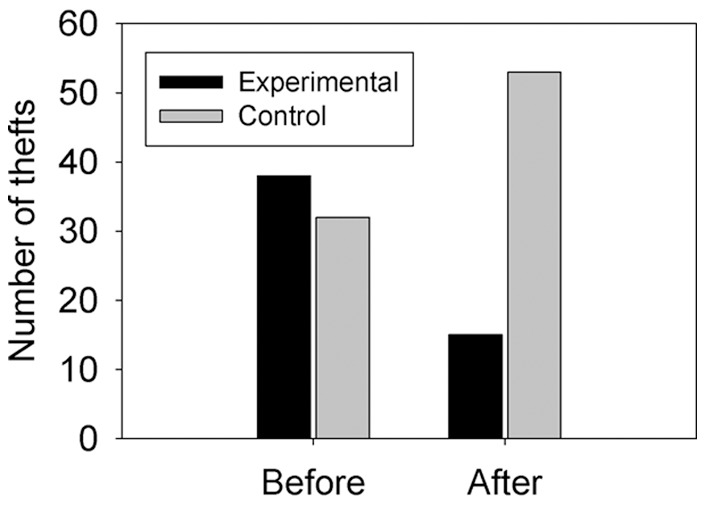
Numbers of notified bicycle thefts in the 12 months before and after the intervention for the experimental locations (black bars) and the control locations (grey bars).

There were 70 cycle thefts notified in the 12 months prior to the onset of the intervention, and 68 in the 12 months following it. Two of the thefts in the 12 months after the intervention did not have a location identified in the database, and are excluded from further analysis. The number of thefts from experimental locations was 39 in the 12 months prior to the intervention, and 15 in the 12 months following it, a decrease of 62%. A decrease in thefts was observed in all three of the experimental locations considered separately (21 vs. 7; 13 vs. 6; 5 vs. 2). In contrast, the number of thefts from control locations was 31 in the 12 months prior to the intervention and 51 in the 12 months following it, an increase of 65%. This represents a highly significant association between intervention and change in number of thefts (Fisher’s Exact Test, p = 0.0001; see Fig. 2). The fact that there was an increase in thefts at control locations almost exactly equal to the reduction at the three experimental locations strongly suggests displacement from the experimental locations to the rest of campus.

In the 12 months prior to the intervention, there were 31 thefts from 16 different control locations. In the 12 months following it, there were 30 further thefts from these locations. In addition, there were 21 thefts from 14 locations which had not had any theft in the 12 months prior to the intervention. Thus, it seems likely that the displacement of crime from the experimental locations was largely to novel locations where there had not been any thefts before. In many cases, these novel locations were within a few hundred metres of the experimental locations.

Walsh and Farrington [6] recommend expressing the effects of place-based crime interventions as odds ratios (O.R.s) of the crime being committed in a control vs. experimental location after vs. before the intervention. Thus, an intervention which has no effect will produce an O.R. of 1, an intervention which increases crime in the experimental locations relative to the control locations will produce an O.R. significantly less than 1, and an intervention which decreases crime in the experimental location relative to the control locations will produce an O.R. significantly greater than 1. Here, using the formulae provided in [6], the O.R. was 4.28 (95% confidence interval 2.04–8.98). This means that the odds of a theft occurring in a control location are increased more than four-fold by the installation of the signs at the experimental locations.

To investigate whether the effect of the intervention signs attenuated over time, we split the post-intervention study period into two 6-month blocks. The number of thefts from experimental locations was 8 in the first 6 months and 7 in the second 6 months. For the control locations, there were 23 in the first 6 months, and 28 in the second. The O.R. for the first 6 months after the intervention compared to the 6 months before it was 4.79 (95% confidence interval 2.28–10.06), and that for the second 6 months was 6.67 (95% confidence interval 3.18–13.9). Thus, there is no evidence of any attenuation of the effect of the intervention over time within the year we studied.

## Discussion

The simple intervention of displaying signs featuring images of watching eyes and a verbal message about being watched was associated with a large reduction of bicycle thefts at the experimental locations, reducing them from 39 in the year before the intervention compared to 15 in the year after. Previous studies of the watching eyes effect in real-world settings have focussed on small acts of generosity [19], [20], or relatively minor infringements of social norms such as putting money in an honesty box, littering or disposing of garbage incorrectly [21]–[23]. We were thus surprised to find an apparent effect on the much more serious, and presumably motivationally different, social norm violation of bicycle theft.

Unfortunately, the reduction was almost exactly offset by an increase in thefts from the rest of the campus, suggesting that the principal effect of the signs was to displace offending from their immediate vicinity. The possibility of displacement has long been raised as a limitation of place-based crime prevention interventions [7], though the evidence is that whilst displacement is often observed after such initiatives, it does not universally occur, and in some cases its opposite, diffusion of benefit, is found [8]. Why this intervention in particular produced such a strong displacement effect – and displaced offences such a short distance - is not clear. The signs were in fixed places with a limited field of visibility, and suggested surveillance of that specific location. Thus, they may have led to the perception that moving out of sight of the signs was a sufficient response. Despite this strong displacement effect, there may be potential for the university to achieve overall reductions by blanket application of the intervention at bicycle racks through the campus.

The effect size (an O.R. of 4.28 using the methods of [6]) was very large in terms of previously evaluated crime prevention initiatives. To some extent this method of calculating the O.R. produces a misleading picture where there is displacement of crime from experimental to control locations. Such displacement increases the numerator of the O.R. whilst also decreasing its denominator, producing a kind of double counting of the effect. Nonetheless, just considering the percentage reduction in thefts after the intervention at the experimental locations (62%) suggests a high level of effectiveness. To put the effect in context, Welsh and Farrington [6] presented a meta-analysis of 41 evaluations of closed-circuit television interventions from around the world. The pooled O.R. from these studies was 1.19, and the O.R. of the individual study with the largest effect was 3.34. Thus, our simple intervention had, by this measure, a larger effect than any evaluated closed-circuit television intervention. This is potentially significant. Existing rational-choice approaches to offending assume, quite reasonably, that it is important to increase the level of surveillance of crime locations in order to make the costs of offending large relative to the benefits, through increased probability of detection [29]. However, the current results, in combination with previous research on watching eyes effects (e.g. [14], [19], [21]–[23]), suggest that to change behaviour, it may be sufficient to engage the *psychology* of surveillance, even if no actual increase in surveillance is occurring. This is because there are relatively automatic, fast brain mechanisms that reliably respond to cues – such as eyes – which over evolutionary time have indicated surveillance, even if those cues in the current environment are completely artificial.

A possible implication for policymakers is that crime prevention initiatives do not always need to involve actual surveillance if they can exploit people’s responsiveness to simple surveillance cues. Closed-circuit television is extremely expensive, accounting for three-quarters of anti-crime expenditure in the UK totalling hundreds of millions of pounds [6]. It also raises concerns about privacy and social impact [30]. How much of the crime reductions which follow the installation of closed-circuit television systems could be achieved by a cheaper intervention similar to that used here is at present unknown. It is quite possible that the effects of the current intervention will attenuate over time (though there was no evidence of this during the 12 months of the study), or would reduce once people learned that there were no other new measures lying behind the signs. Thus, one economically attractive possibility would be large-scale use of a cheap sign-based intervention similar to the one used here, combined with probabilistic actual surveillance, to reinforce the perception of being watched with occasional evidence that this perception is real. Where actual surveillance using closed-circuit television is undertaken, its impact might be enhanced by making sure that the psychology of surveillance is engaged as fully as possible, such as by adding eye images and appropriate verbal messages to cameras and making them as conspicuous as possible.

This study was on a relatively small scale compared to other crime-prevention initiatives, and was constrained by what was practicably implementable within our single campus with the information and resources we had. As a result, there are important limitations that should be clearly acknowledged. The first is that there was no replication across stimuli. The signs at all three of our experimental locations were identical, and even if they had been different, statistical power for establishing differential effectiveness would have been very low. Thus, all we can really conclude is that *this* sign has an impact on bicycle theft. We cannot tell which features are critical to its impact or how broadly this generalizes across possible variations on sign design. However, in our previous studies of the watching eyes effect, we have used multiple different eye images and concluded that the observed effects generalize across these [15], [21], [22]. A related point is that our current design did not separate out the effects of merely installing any sign at all from the contents of these signs, or, within the contents of these signs, separate out the effects of the verbal and the image components. Verbal messages alone can have large effects on theft [28], whilst one of our previous studies suggests that watching eyes can improve compliance with social norms even when not displayed with any relevant verbal message [22]. Thus, an important follow-up question would be to establish whether the same effect found here could be achieved with just eye images and no accompanying verbal content, or just a verbal message with no eye images.

Another class of limitation stems from our having no information about how many different people are responsible for bicycle thefts on the campus. This is a common situation in experimental studies of crime prevention interventions, where the number of offences is the outcome variable, and almost by definition the perpetrators are unknown to the researchers. At one extreme, there may be many individuals who independently set out to commit bicycle thefts, in which case our data suggest that the effects generalize across individuals. At the other possible extreme, there may be one small group of thieves whose behaviour has been strongly affected by our manipulation, leaving us unable to be sure that the impact will generalize to other such groups. Unfortunately, we have no information on this point, though we suspect that there are many different thieves. This is relevant to the lack of attenuation of the impact of the signs over time. If the same individuals come back again and again, the lack of attenuation would suggest that individuals do not habituate to the signs. On the other hand, if there are many different individuals each of whom comes to campus on a single occasion, then the lack of attenuation is to be expected, since the signs are always new to the individual even if not new to the campus.

Our study also shed no light on what level of cognitive processing was involved on the part of potential bicycle thieves. On the one hand, it is quite possible that there was deliberate thought involved, with potential offenders reasoning that if there were signs, there might also be other measures such as extra patrols or cameras. If this is the case, then we might expect that the effectiveness of the signs will attenuate with time or once the details of the intervention become more widely known about, and, unfortunately, greater public awareness of the watching eyes phenomenon through this and other publications may actually diminish its effectiveness. On the other hand, the effect may largely occur at a more implicit and automatic level. Some previous studies of the watching eyes effect have suggested that people exposed to watching eyes do not report feeling any less anonymous or more observed when asked, even though their behaviour is different [15], [23]. If this proves to be the case, the effect of this intervention could be relatively resistant to habituation or to explicit knowledge about security policies or the watching eyes effect.
